# Supplementary material for: Modeling nearshore-offshore water exchange in Lake Ontario
Source: PLoS One. 2025 Jan 8;20(1):e0298702. doi: 10.1371/journal.pone.0298702 (PMC11709247; doi:10.1371/journal.pone.0298702)
Supplement: Attachment — Submitted filename: RebuttalLetter.pdf [file pone.0298702.s002.pdf]

Bogdan Hlevca
Ontario Ministry of the Environment,
Conservation and Parks
125 Resources Road, Toronto, Canada
E-mail: bogdan.hlevca@ontario.ca
bogdan@hlevca.com
September 17th, 2024

Dr. Joao Miguel Dias
Editor-in-Chief
PLOS ONE

Dear Dr. Dias,

I am pleased to submit a revised version of the original research article entitled "*Modeling nearshore-offshore water exchange in Lake Ontario*" by Bogdan Hlevca, Todd Howell, Reza Valipour and Mohammad Madani for consideration for publication in your journal *PLOS ONE*. We confirm that this manuscript has not been published elsewhere and is not under consideration by another journal. All authors have approved the revised manuscript and agree with its submission to the *PLOS ONE* journal.

We strived to respond to all reviewers' comments and revise the manuscript accordingly. We hope that the new version of the manuscript has addressed all the reviewers' concerns.

Yours truly,

Bogdan Hlevca

Response to reviewers

Reviewer #1:

Review of "Modeling nearshore-offshore water exchange in Lake Ontario".
The manuscript describes the exchange dynamics between near- and offshore areas of Lake Ontario based on numerical simulations. The topic is interesting, and the methodology followed is efficient. However, there are several points that require more clarifications and changes. I suggest major revisions based on my comments below.

A: We thank the reviewer for the appreciative comments and suggestions for improvement.

General comment 1: The vertical structure of the water column is not described at all. Only surface and deep temperature is shown. E.g., stratification frequency is presented only in supplementary material, but it could be very useful to be included in the main manuscript.

A: We designed this manuscript as a follow up to another manuscript that was submitted for publication, which is extensively describing the water column. We felt that the focus of this manuscript should be on the nearshore-offshore water exchange and keep to the minimum the information leading to the exchange. We added the relevant information in the Supplementary document for those interested to know more about the lake conditions. However, we understand the point of view of the reviewer that a complete information would be providing a better picture of the state of the lake without needing to do more research. In the revised version we added the information requested including one figure, new Figure 2.

General comment 2: The authors use the term "mixing" to express the horizontal advection or dispersion of tracers. Mixing usually refers vertical mixing. I suggest to correct this throughout the text.

A: We agree with the reviewer that lake mixing is generally referred by most authors in relation to the lake turnover. However, in the general sense of the word, mixing is related to the transport of matter in a fluid (here, the exchange of water between two parts of the lake), where the matter may be dissolved in the fluid. Also, mixing may also occur in connection with the transport of momentum and energy, irrespective of the direction. Mixing as a generic term implies that the transport reduces or eliminates differences in space and time, between fluid elements with different content. We understand the reviewer's concern and we think that since the manuscript is very specific about the exchange between the nearshore and the offshore, no confusion is possible. Where possible, we replaced mixing with dispersion or qualified it with "horizontal".

The term mixing is used by other researchers, for example reference #24 & #26 from bibliography:

> **Rao YR, Schwab DJ. Transport and mixing between the coastal and offshore waters in the GreatLakes: a review. J. Gr. Lakes Res. 2007; 33:202–218.**

Jabbari A, Valipour R, Ackerman, JD, Rao YR. Nearshore-offshore exchanges by enhanced turbulent mixing along the north shore of Lake Ontario. J. Gr. Lakes Res. 2023; 49(3):596-607.

General comment 3: The information about the different experiments/simulations is a bit confusing. The descriptions should be clarified (maybe with a use of a table?) and details about the runs' differences (and the purpose for each simulation) should be included. E.g., I am confused about the release areas of tracers in the first and second simulation studies. Moreover, more information about the type and characteristics of the Lagrangian simulations is needed.

A: We have rewritten the chapter "Modelling scenarios and definition of tracers" to make the information flow clearer. We also added a table (Table 1) with the simulations and what tracers have been used in each simulation. As a note, we have not used Lagrangian modelling, please see the explanation at the next point. The tracers were released at 8 locations around the lake, and we used the Eulerian transport equations to describe their evolution.

General comment 4: It is not clear, at least to me, why the authors chose to perform another set of simulation with the offshore boundary conditions and did not use the typical set up with releases at the 8 locations. This is maybe a problem of the simulations description (see above), which is a bit confusing.:

A: The description of the simulation scenarios and the description of the tracers has been updated and partially rewritten. We added a table with tracers and the simulations to improve clarity. The NRT tracer, released at 8 locations has been used to determine the horizontal dispersion whereas the offshore constant boundary condition was used to determine water age. Since our modelling framework does not allow to set constant values inside the computational domain, an offshore domain has been defined to just do that.

The tracers had different purposes. The age tracer is self explanatory and the point regional tracer NRT was used to estimate along-sore and cross shore dispersion, while the NST was used to measure the nearshore-offshore exchange. NST is a whole body of water tracer, but both NST and NRT use Eulerian transport equations. There was no Lagrangian simulation performed in this study. We have rewritten the chapter that describes the tracers and the simulation to clarify any confusions induced in the first version of the manuscript.

Several specific comments (not in an order of importance) are provided below.

Specific comments

Chapter numbering is missing.

A: The decision not to number the chapters was deliberate as none of the articles published in PLOS ONE, we looked at had section numbering. We followed the rules that we thought the journal endorses.

L31: north or south?

A: As the climate warms, northern regions that were before too cold for certain species become suitable for warmer water species. Therefore, their habitat is extending toward north.

Hlevca et al. paper that presents the set-up and validation of the model used here is not yet published. It should be replaced with the published when/if it is published.

A: The paper is under review with minor changes requested. The reference will be changed as if and soon as the manuscript will be accepted.

Fig. 1: A point at the westernmost tip of the lake does not have name.

A: We thank the reviewer for pointing that out. The GIS tool might have removed the label due to overlapping. The label issue is now fixed.

L113-114: Please explain.

A: Models can be set with constant boundary conditions, but one cannot impose constant values inside the computational domain in the MIKE 3 modelling framework. We modified the text for clarity.

Simulation of age: It is confusing to understand which of the two simulation studies is used here. I guess it is the second one, but it should be clarified for all results. Please see also previous comment about the simulations' description.

A: We have updated and partially rewritten the chapter that describes the tracers and simulations. We added a table to add more conciseness. Age and NRT tracers were modelled in the first simulation. We apologize for creating confusion in the first version of the manuscript. The combination of tracers and assigned simulation is complicated and difficult to describe. We hope that the new version is easier to understand.

L152: I can’t see that. I see strong variation but not increasing trend after 135.

A: There is a strong variation but overall, there is still a non monotonous increasing trend in both 3 and 30 m depth to at least DOY 235 (end of August). We made the correction.

L161-162: increase maybe? Shaded area cover a period of higher ages, no?

A: Thank you for catching this error. We made the correction.

L168: I do not understand or even see this in the figure.

A: We noticed several lines below you had a question *“-What is this “strange” dashed line right next to the left y-axis?”* We documented this line in the Fig 3 caption “The black dash-dotted line is representing the ‘no-exchange’ aging line.” This is what it was referred at line 168 in the first submission.

Fig. 2: Figure and caption need corrections. Labelled points? “The nearshore points of release are located at a water depth of less than 30 m.”. Are these presented in Fig. 2? Vertical transect?

A: We thank the reviewer for noticing this error. The text from Figure 1 caption was carried over. From the previous figure and not corrected. We apologize for this error. The caption is now corrected with the proper text.

Fig. 3:
- Please put legend in (b) and include all lines (temperature and ages) in both panels’ legends.

A: The figures were too busy to include all the plots in the legend. We created a legend on the side of the figure, which is now Figure 4.

- Why not keeping the same color as age for temperature?

A: We though of making them more visually distinct and easier to refer to them in the text. However, we have revised the color scheme and colors are consistent in all figures.

- Please align the two panels to facilitate viewing the changes in time between timeseries

A: We made the x-axes equal, as requested

- It is minor but why does the time x-axis stop a few days after 300 and not incudes the entire year?

A: The available field data ends at the beginning of November and we used the same time interval for the data generated by the model for this particular figure. The dates outside this interval have no relevance to the analysis.

-What is this “strange” dashed line right next to the left y-axis?

A: the “strange” dashed line in Figure 3a is documented in the legend and it is also explained in the figure caption. It represents the water age values in the absence of mixing, i.e., how the water would age in the nearshore if no mixing with the offshore water would take place.

- Table 1: Ages can also be included with a separate column, and it should be described more in the text.

A: Table 1 (now Table 2) describes the upwelling and downwelling events and although they influence the water age they cannot be put together in the same table. We expanded and clarified the description of the water age in the text.

- Simulation of nearshore regional tracers: So, are we still in the first simulation? It was stated that this simulation starts in April…How can we see results for entire year in Fig. 3? Please see previous comment about the simulation’s descriptions. Clarifications about the simulations/periods and which version is used for what reason/results is needed!

A: We understand that the description of the simulation was confusing as they involved sometimes 2 tracers. All the simulations start in January, while the releases of the tracers were started at different dates to capture the difference in water movement across the seasons. We clarified this in the text and added a new table (Table 1) that describes the relationship between the tracers and simulations.

L198: Niagara is west or south?

A: we thank the reviewer to catch this inconsistency. Indeed, Niagara is located in the south-west of the lake and does not meet the description for northern and western sites, unless we consider Niagara as western only. We made the change to be clearer.

L203: This is just an example. Please mark all topographic features, mentioned in the text, in figure 1.

A: We added in Figure 1 all the topographic names that are referred in the text.

L213: Same here

A: We added in Figure 1 all the topographic names that are referred in the text.

Fig. 4: L223-224: Which intervals? Averages for 62 days?

A: The text was ambiguous. We made changes to the text stating that the vectors show the direction of the current at the end of the time interval (62 days). Now is Fig 5.

In all figures: use dates instead of only numbers. It is getting confusing (e.g., Fig. 5)

A: Most of the limnology papers use day of the year (DOY) for many reasons including saving space and declutter. We believe that used DOY is a reasonable approach with little inconvenience. We agree that a quick look may lead to confusion in Figure 5 (now Figure 6). However, the x-axes are labelled for the bottom panels of the figure, and it should prevent confusion.

L250: This is the case in all scenarios. Please comment.

A: We made some changes in the text to increase clarity, but the explanation for the suggested line is already given in the following sentence, which explains that the slower mixing in the hypolimnion can be attributed to the presence of a strong thermocline during the stronger stratification period (June-September). While the phenomenon is present in all the scenarios in the scenario where the tracer is released in June the visual separation is much stronger.

Fig. 5: Averaged over which areas exactly? Not sure what the shaded areas show.

A: The meaning of the shaded area is described in the caption of every figure that has the feature. The shaded area covers the time interval where the average age is the highest.

.
L269: “a single year”. And even faster for the surface layer, no?

A: Yes, faster for the surface layers. We added the note in the text.

L271: “discussed above”. Where? In Fig. 3? Fig. 5 does not present ages.

A: We added the reference to the figure to avoid confusion.

Fig. 6: Please mark with a title on the panels the respective locations.

A: Figure 6 (now Figure 7) has the locations documented in the caption. We think that adding location names in the figure would add clutter to an already very busy figure, which has marked the events at the top where the title would have been printed.

Table 2. Hamilton is missing in Fig. 1.

A: Figure 1 has bee modified to label all the topographic features including the missing label of the tracer, which was initially hidden by the automatic placement of the GIS tool.

Fig. 7. r=-0.22 in 7c does not agree with the r in 7d. Please check.

A: We checked. The values in the left panels of the figure are calculated by the algorithm and are accurate. The figures ion the right are calculated though windowing and may show same small discrepancies as the window boundaries may not fall exactly on the maximum/minimum value, but are in general agreement with the values on the left. In this case is r= -22 on the left and around -20 on the right panel. The same small differences can be observed in the other panels too. We think that the important message given in the right panels is that the wind and temperature are the determining factors and lead the changes in *age.*

Fig. 8. Discussion about this figure is nearly absent. More details are needed. What about the rest of the year that does not have any values in (b).

A: The figure and the topic were last-minute additions, and we did not want to make the manuscript too long and we put brief explanations in the figure caption. However, we agree with the reviewer that o more thorough discussion is needed. We added comments in the text to make the information related to the figure clearer. The rest of the year in panel b (hypolimnetic flux) and c (hypolimnetic contribution %) the exchange is not present because there is no thermocline and therefore there is no distinction between epilimnion and hypolimnion.

L343: "2 passive tracers". Two sets? Two tracers?

A: We thank and agree with the reviewer that formulation of "2 passive tracers" is not correctly describing the study. There is only one tracer, in this case NRT, which is released from 8 locations. We have made the change in the text.

L353: open…open

A: We noticed the repetition and corrected the text

L381-383: This conclusion is not supported by the results. Please explain or remove.

A: We thank the reviewer for the comment. When adding the text to explain fig 8 (now Fig 9) we also added the explanation for this statement. We also made corrections in the text to add clarity.

L386-388. Same here.

A: That sentence was a speculative/logical assumption based on the hydrodynamics and it is not directly supported by the results. Since is not essential to the study, we removed it.

Reviewer #2:

In this manuscript, the authors used a three-dimensional hydrodynamic model along with a series of virtual tracer numerical experiments to investigate the water exchange between the nearshore and offshore areas of Lake Ontario. They found that cross-shore transport varies across different nearshore regions and is more pronounced during the stratification period than the water turnover period. Additionally, coastal upwelling events can contribute to cross-shore transport. These findings are meaningful as they enhance our understanding of nutrient transport and its connection to algae growth in the nearshore region.

However, the results and conclusions do not seem entirely convincing to me, as the manuscript lacks discussion on many aspects. It is also somewhat frustrating that all conclusions are based on simulations spanning only one year. Given these considerations, I recommend a major revision.

A: We thank the reviewer for the appreciative comments, and we made changes to the manuscript to address the issues that were raised by the reviewer.

Major comments

1. One aspect that I think the study lacks discussion on is the impact of weather-scale wind events on cross-shelf transport in the top layer. Given the relatively shallow depth of the nearshore region, the magnitude and speed of weather-scale wind events can significantly influence its cross-shelf transport. For instance, Figure 4a shows strong alongshore dispersal in the northern nearshore, which the authors attribute to the cyclonic spin in the upper Mississauga Basin. However, it is possible that around DOY 62, before and after, a strong south wind occurred and inhibited water movement in the northern region. I think that an analysis of longer-term average currents and concentrations, along with a discussion on their variations in relation to weather-scale wind events, is necessary for a more comprehensive understanding.

Thank you for your comment. Indeed, long-term studies are required to better characterize these events. Unfortunately, we only had a limited set of field data for year 2013 and we used the 2018 data to validate the model. To address this issue, we have added this paragraph in the discussion section:

"Coastal upwelling events significantly influence seasonal nearshore-offshore exchanges. For instance, during upwelling events #4 and #10 (Fig. 2), depth-averaged cross-shore velocities of approximately 0.014 m/s and 0.019 m/s, respectively, would result in cross-shore excursions of approximately 1.1 km and 1.7 km per day along the north shore, assuming horizontal transport is primarily driven by advection. Accurately assessing historical trends in nearshore-offshore exchanges necessitates long-term records of water temperature and wind dynamics in coastal regions. In this study, we present an estimate of nearshore upwelling in Lake Ontario based on the data acquired during the field season, which was also used for the calibration of the model. The observed increasing trend in the frequency of upwelling-favorable winds along the north shore of Lake Ontario aligns with recent findings that indicate a rise in extreme eastward wind events facilitating inter-basin exchanges in Lake Erie [38]. Further investigation into similar wind-induced events is essential for predicting intra-basin and inter-basin physical and biological processes in lakes and coastal oceans."

Also, in our study we noticed that enhanced vertical mixing events caused by coastal upwelling events in northern Lake Ontario. When such coastal upwelling events occur, they could temporarily change the nearshore nutrient (i.e., SRP) dynamics and availability through offshore-nearshore exchange. Our model results show that such elevated turbulent dynamics indicate higher potential for nutrient mixing. When offshore hypolimnetic waters are enriched with nutrients, coastal upwelling events can transport them into the nearshore waters.

2. I was wondering, did the hydrodynamic simulation include ice? If so, it is very interesting to see an enhancement of the cross-shore transport starting at DOY 30 in Figure 6a, coinciding with the time when ice begins to form more. Can you explain it a little bit? If not, please clarify it in the manuscript, as I suppose the nearshore-offshore water exchange will be very different in water with and without ice.

A: The simulation did not include ice as the 2018 was an ice-free year. The year 2013, which was used for calibrating the model (documented in a sister study/manuscript) has ice coverage, but we have not used 2013 data for this modelling study. We think that the cross-shore transport the reviewer is referring to has more to do with the warming period of the model since its initial conditions are 4 C for temperature for the entire water column and zero for velocity.

We added the following sentence at the end of the Methods section: "*The simulations did not include ice coverage as 2018 was an ice-free year for Lake Ontario, with a short-lived exception in the Kingston Basin where partial ice-coverage lasted for a few days in mid-January".*

3. In the summary and conclusion, it is glad to see the author connecting the virtual tracer experiments with the nearshore shunt hypothesis regarding the nuisance conditions of Cladophora in the Lake Ontario nearshore area. The tracer experiments, suggested by the younger age indicators, point to dilution by offshore water, which transports particulate phosphorus (PP) to the nearshore area for mussel filtration. However, one aspect the author has overlooked is the simultaneous transport of soluble reactive phosphorus, excreted by mussels, to the offshore region through nearshore-offshore water exchange. This dual flux—water moving from offshore to nearshore and from nearshore to offshore—is typically not well balanced for individual cells or small regions. Consequently, in some areas, Cladophora may not grow to nuisance levels if the nearshore-offshore transport is significantly strong. Could the author elaborate more on this?

A: We totally agree with the reviewer that mussels play an important role in the phosphorus cycle in the nearshore. We developed and calibrated a MIKE Eco Lab template that implements the Nearshore shunt theory. Our next goal is to explore in detail the interplay between nutrients Cladophora and Dreissena mussels This paper, however, focused on the nearshore-offshore exchange and we hypothesized that the offshore nutrients could have an important contribution to the balance that is often ignored.

4. I think the term "mixing" as used in the manuscript is ambiguous and requires clearer definition. In most instances within the manuscript, it seems the authors are referring to the "mixing" as the integration of nearshore and offshore waters, primarily driven by horizontal advection. However, in several sections, "mixing" is used to denote diffusion processes.

A: We thank the reviewer for pointing to these inconsistencies. We have corrected the use of mixing to avoid ambiguities and our term mixing is qualified and mostly refers to horizontal mixing and dispersion. We did not refer in our study to diffusion, which is a microscopic phenomenon and mostly used in reference to molecular motion. Here we only study macroscopic phenomena caused by non-ideal flow patterns.

Also, please note that the term mixing is used by other researchers, for example reference #24 & #26 from bibliography:

> **Rao YR, Schwab DJ. Transport and mixing between the coastal and offshore waters in the Great Lakes: a review. J. Gr. Lakes Res. 2007; 33:202–218.**
>
> **Jabbari A, Valipour R, Ackerman, JD, Rao YR. Nearshore-offshore exchanges by enhanced turbulent mixing along the north shore of Lake Ontario. J. Gr. Lakes Res. 2023; 49(3):596-607.**

Minor comments

1. I suggest adding one or two figures illustrating Lake Ontario's climatological circulation patterns and their seasonal variations in the introduction section to give readers a better understanding.

A: We appreciate the comment. However, this study focuses on the lake responses to meteorological forcing using publicly available datasets. We have sister study related to the current one, which is also under review, which studies the lake response to climatological forcing. This study is referred in the bibliography: https://www.researchsquare.com/article/rs-3296977/v2

2. Lines 95-98: What about the water balance for the cell or grid? Can it cause an artificial plume in the cell and adjacent cells?

A: The water balance is preserved in the MIKE 3 modelling framework and the "MIKE 3 Flow Model FM Hydrodynamic and Transport Module Scientific Documentation (e.g., page 33), which can be found at: https://manuals.mikepoweredbydhi.help/latest/Coast_and_Sea/MIKE_3_Flow_FM_Scientific_Doc.pdf

Artificial dispersion can be caused by suboptimal meshes. For example, in models with steep bottoms that use Sigma coordinates artificial vertical velocities induce numerical vertical dispersion. Our model was carefully calibrated for minimal digital artifacts.

3. Line 224: There are no average currents shown in Figure 6.

A: Thank you for noticing the inaccuracy. The text was pointing to a figure that was subsequently deleted, but the reference was not. The reference has been now removed.

4. Please add those stations or places (e.g., Oswego region, Humber Bay) to Fig. 4. Otherwise, it becomes difficult for readers to follow what you describe in the results section.

A: Thank you for you comment. We have all this information provided in Fig 1. While it may be inconvenient for readers that don't know the topography of the lake, we think that keeping the figure emphasized on presenting the processes uncluttered is more beneficial to the reader.

5. Lines 340-342: Lake Ontario is a dimictic lake. The water stratifies in winter and summer and is well-mixed in spring and autumn. Currently, the lake is not well-mixed during winter under the current climatic conditions….

A: Thank you for spotting the error, it was a typo, we mistyped "winter" instead of "fall". It is corrected now.
