# Supplementary material for: Modeling nearshore-offshore water exchange in Lake Ontario
Source: PLoS One. 2025 Jan 8;20(1):e0298702. doi: 10.1371/journal.pone.0298702 (PMC11709247; doi:10.1371/journal.pone.0298702)
Supplement: Attachment — Submitted filename: RebuttalLetter_R2.pdf [file pone.0298702.s003.pdf]

Bogdan Hlevca
Ontario Ministry of the Environment,
Conservation and Parks
125 Resources Road, Toronto, Canada
E-mail: bogdan.hlevca@ontario.ca
bogdan@hlevca.com
October 27th, 2024

Dr. Joao Miguel Dias
Editor-in-Chief
PLOS ONE

Dear Dr. Dias,

I am pleased to submit a second revised version of the original research article entitled "*Modeling nearshore-offshore water exchange in Lake Ontario*" by Bogdan Hlevca, Todd Howell, Reza Valipour and Mohammad Madani for consideration for publication in your journal *PLOS ONE*. We confirm that this manuscript has not been published elsewhere and is not under consideration by another journal. All authors have approved the revised manuscript and agree with its submission to the *PLOS ONE* journal.

We strived to respond to the remaining minor issues flagged by the reviewers and revised the manuscript accordingly. We hope that the new version of the manuscript has addressed all the reviewers' concerns.

Point by point responses are given below for the last two remaining issues.

In addition to those changes, reference #30 has been modified from preprint to published.

Yours truly,

Bogdan Hlevca

Response to reviewers

Reviewer #2: The authors have well addressed all my comments. Good job! I recommend this paper for publication. However, two minor things should be awarded before final publication:

1). The figures in the revised version are low resolution, making some labels difficult to read. Please ensure that high-resolution figures are included in the published version.

A: All the figures were submitted in vey high resolution as per the following table:

#	Size W x H	DPI
Fig1	3507x2480	300x300
Fig2	2800x2800	300x300
Fig3	3447x1661	300x300
Fig4	3000x2781	300x300
Fig5	1906x2340	300x300
Fig6	1276x1426	300x300
Fig7	2250x2327	300x300
Fig8	1325x1665	300x300
Fig9	1907x1370	300x300

We believe that the reviewer referred to certain figures that appear to have a small font because the resolution is too high and when rendered on screen or inside a document, including in the PDF reader, the images are shrunk, which makes the font to appear smaller.
We have identified the figures that were generated at a resolution that was too high for the accepted standard and regenerated them by adjusting the sizes and also used larger fonts. The PLOS ONE file requirements that were followed are the following:

- Width: 789 – 2250 pixels (at 300 dpi). Height maximum: 2625 pixels (at 300 dpi).
- Maximum width: 19.05 cm (7.5 in), 2250 pixels
- Maximum height: 22.23 cm (8.75 in) 2625 pixels

The following figures have been regenerated (please note that Fig 2 from above is now Fig 3 and Fig 3 from above is now Fig 2):

#	Size W x H	DPI	Redone: Yes/No
Fig1	2250x1687	432x432	Yes
Fig2	2250x1084	300x300	Yes
Fig3	2250x2400	300x300	Yes
Fig4	2250x2100	300x300	Yes
Fig5	1906x2340	300x300	No
Fig6	1276x1426	300x300	No
Fig7	2250x2327	300x300	No

Fig8	1325x1665	300x300	No
Fig9	1907x1370	300x300	No

2). Fig. 2 and Fig. 3 should switch positions, as Fig. 3 is mentioned first in the Methods, while Fig. 2 is first mentioned in the Results."

A: We thank the reviewer for noticing this error. We have reversed the 2 figures names, and their references in the text, including the captions.
